# Carbon isotope effects in the sorption of chlorinated ethenes on biochar and activated carbon

**DOI:** 10.1016/j.heliyon.2023.e20823

**Published:** 2023-10-10

**Authors:** Simon Leitner, Fridjof Sobanski, Gerhard Soja, Katharina Keiblinger, Christine Stumpp, Andrea Watzinger

**Affiliations:** a University of Natural Resources and Life Sciences, Vienna, Austria; b Institute of Soil Research, Konrad-Lorenz-Straße 24, 3430 Tulln, Austria; c University of Natural Resources and Life Sciences, Vienna, Austria; d Institute of Soil Physics and Rural Water Management, Muthgasse 18, 1190 Vienna, Austria; e AIT Austrian Institute of Technology, Konrad-Lorenz-Straße 24, 3430 Tulln, Austria; f University of Natural Resources and Life Sciences, Vienna, Austria; g Institute of Chemical and Energy Engineering, Muthgasse 107, 1190 Vienna, Austria

**Keywords:** Biochar, Stable isotopic fractionation, Sorption, Biofilter, Chlorinated ethenes, Groundwater purification

## Abstract

As an alternative to activated carbon, biochar is a promising, environmentally friendly sorbent that can be used to remove organic groundwater pollutants, such as chlorinated ethenes (CEs). Stable isotope fractionation in biofilters is used to quantify pollutant degradation and to distinguish degradation from pollutant sorption on e.g. biochar. However, the sorption of CEs on biochar, and the potential abiotic fractionation processes remain to be tested. The sorption process of CEs and ethene on activated carbon and biochar was investigated with regard to the isotope effects for the differentiation from microbial degradation processes. Results from physical and chemical characterization of biochar indicated that biochar feedstock and pyrolysis conditions determined sorption performance depending on the surface chemistry and the pore size distribution of the coarse sorbent particles. The sorption capacity of the activated carbon was significantly higher with highly chlorinated ethenes, but similar to the biochars with low chlorination. Apparent carbon isotope fractionation factors (ε) of +0.1 to −4.4 ‰ were found above measurement uncertainties of GC/IRMS. The extent of isotope enrichment of the ^13^C bearing isotopologues in the residual aqueous phase (ε < 0) was characteristic for individual pairs of pollutant and sorbent material and could be related to pore-filling processes limited by the micropore size distribution of sorbent materials and the chemical properties of sorbed pollutants. Especially the large isotope fractionation during the sorption of ethene led to the assumption that diffusion processes within the pore matrix of the sorbent particles contributed to the observed isotope effects, but should still be considered a property of sorption. Concluding on the results indicated that sorption processes can have a significant contribution to carbon isotope fractionation in CEs and ethene. These should not be neglected in the evaluation of biofilters for groundwater purification, in which CEs are simultaneously degraded by microbes.

## 1 Introduction

Porous carbonaceous materials such as activated carbon (AC) can efficiently remove organic pollutants from gases and liquids by sorption processes. This concept is frequently applied for the remediation of contaminated groundwater. Given the chemical and energy requirements involved in producing AC, alternative sorbent materials such as biochar (BC) have been attracting considerable interest. These can be produced from numerous waste products from agriculture and forestry and represent a more cost-effective and environmentally friendly alternative and have already been tested with the common groundwater contaminants tetrachloroethene (PCE) and trichloroethene (TCE) [[1], [2], [3], [4], [5]].

From an economic point of view, in addition to the costs for a suitable sorbent material, a long maintenance-free filter life is of interest. One approach to extending the service life, that has already been tested, is the use of contaminant-degrading microbes, which colonize the surfaces of the sorbent and thus counteract the depletion of the sorption capacity (e.g. Refs. [[6], [7], [8]]). For the evaluation of this combined approach, it is necessary to differentiate compound and metabolite transformation and dispersion (e.g. biodegradation and sorption), whereby the quantification of any degradation products is not necessarily sufficient. A valid method for clearly separating compound degradation and sorption is compound specific stable isotope analysis (CSIA). The stable isotope ratio of, for example, carbon in the contaminant molecules can provide information on whether these substances have been modified by biological, chemical or physical processes [9]. As an example, ^13^C-bearing isotopologues of chlorinated ethenes (CEs) showed an accumulation in the aqueous phase during microbial degradation [10], while their evaporation showed an inverse isotope effect [11,12]. The isotope effect in compound degradation is caused by differences in activation energies that favour turnover of less mass heavy isotopologues (normal kinetic isotope effect) [13].

Suppression of the horizontal and vertical zero-point vibrational energy in a molecule at low temperatures would lead to an isotope effect expressed by favourable sorption of the less mass heavy isotopologues on the sorbent surface [14]. On the other hand di Corcia and Liberti (1970) [15] observed the opposite, reasoned by the more compact electron distribution leading to a lower polarizability of heavier isotopes and isotopologues. This caused the resulting more distant electron orbitals of lighter isotopes to move the entire molecule closer to the sorbent surface. So far, the impact of sorption processes in changes of the stable carbon isotope ratio of CE have been considered marginal or negligible [11,12,16,17]. The identification of apparent isotope fractionation at the sorption equilibrium depends on the underlying analytical measurement uncertainty (∼0.5‰) for continuous flow stable isotope analysis using gas chromatography coupled to stable isotope ratio mass spectrometry (GC/IRMS) [18]. Sorption equilibrium is predominately controlled by the Gibbs energy and isotope effects are of minor importance [19]. Nonetheless results from previous studies suggested that sorption of CEs onto carbonaceous sorbent materials lead to an isotope effect with an accumulation of ^13^C-isotopologues in the aqueous phase and ^12^C isotopologues on the adsorbent [16,20,21]. Such an isotope effect was also detected for other chlorinated hydrocarbons [22], but was always within the measurement uncertainty of 0.5‰. Slater et al. (2000) [16] stated that the sorption-related isotope effect for PCE and TCE could exceed the measurement uncertainty of 0.5‰ if the sorbed mass fraction were to exceed 95%. This hypothesis was taken up by Schüth et al. (2003) [21] and rejected on the basis of their result with powdered charcoal particles. However, Kasozi et al. (2010) [5] showed that the sorption properties are altered by the sorbent particle size. Furthermore, Li et al. (2001) [23] demonstrated that the sorption capacity is also influenced by the kinetic diameter of the adsorbate and the matching pore size distribution of the sorbent material. Kopinke et al. (2005) [24] conducted a multistep experiment, where the solvent was repeatedly exchanged after reaching the sorption equilibrium in order to observe isotope fractionation processes in the sorption equilibrium. The authors concluded that scenarios could be constructed in which sorption-based isotopic effects could compete with chemical or microbial degradation based fractionation. In addition, Wanner et al. (2017) [22] already reported sorption-related isotope effects for other chlorinated hydrocarbons with a shift in carbon isotope ratios of ∼2 ‰.

Isotope effects are most apparent at low residual fractions of a compound in the solvent [19], high loading of the adsorbent (decrease in adsorption enthalpy with increasing loading of the adsorbent) [25], and an ideal pore size distribution of the sorbent material in regards of the adsorbate molecule. We therefore hypothesized that the extent of isotope effects in the sorption equilibrium depends on the chemical and physical properties of the adsorbent/adsorbate pair, as long as the sorption processes are driven by van der Waals forces. Following the idea of the multistep experiment proposed by Kopinke et al. (2005) [24], we hypothesize that a low residual compound concentration in the solvent and a high loading of the coarse-grained sorbent might make a sorption-specific equilibrium isotope effect detectable.

In order to test this hypothesis, biochar (BC) was produced and characterized together with an activated carbon (AC) with regard to their physical and chemical properties, and stable carbon isotopes of sorbates were analysed during sorption experiments. Kinetic and sorption isotherm experiments using single-solute and multi-solute approaches comprising of mixtures of chlorinated ethenes (CEs) PCE, TCE, *cis*-dichloroethene (cDCE) and vinyl chloride (VC) and ethene were conducted. In this way, the mother compound and metabolites produced during anaerobic microbial degradation of CEs were targeted in order to allow conclusions on biofilters for groundwater purification using biochar and microbes with regard to sorbate competition/availability and synergy effects. To our knowledge, this is the first study investigating apparent isotope effects for the sorption of CE and ethene (both expressed as ET) on different sustainable carbonaceous sorbent materials. The results should give a better understanding of the biochar effects in remediation measures and discuss the possible extent of carbon isotope fractionation of ET during sorption processes and the resulting implications for the evaluation of groundwater remediation measures.

## 2 Materials and methods

### 2.1 Biochar production and characterization

Biochar was produced from four feedstocks (apricot kernels (A), wood chips (W), sunflower seed shells (S), miscanthus grass (M) (provided by local farmers)) and at four temperatures (350 °C, 550 °C, 650 °C, 750 °C) using a Pyreka 3.0 pyrolysis reactor (Pyreg GmbH, Dörth, Germany). The raw materials were pyrolysed under a stream of nitrogen gas (0.005 m³ min-1) for 20 min. The biochars were analysed by Eurofins Umwelt Ost GmbH (Freiberg, Germany) for their elemental composition and general physical/chemical properties, including the parameters of the European Biochar Certificate (EBC, [26]) guideline. Carbon and nitrogen content of the feedstocks were determined in-house using an elemental analyser connected to an IRMS (Flash 2000 CHNS/O Analyser, Thermo Scientific, Cambridge, UK; DeltaV Advantage, Thermo Scientific, Bremen, Germany). In-house analyses and experiments were conducted using wet sieved sorbent materials (fraction of 0.63–2.0 mm) to avoid sorption to colloids, which impact sorption capacity [27], thus biasing sorbent comparison.

Sorbent materials comprised of the produced biochars and were supplemented by an activated carbon (AC, Hydraffin A 4x8, Donau Carbon GmbH, Frankfurt am Main, Germany). Screening of functional groups on the surface of sorbent materials was done by Fourier transform infrared (FT-IR) spectroscopy of the ground particles with attenuated total reflection mid-infrared (ATR-MIR) spectroscopy using a Tensor 27 instrument (Bruker, Austria). Spectra were recorded for the 4000–400 cm-1 wavenumber range with five measurements of 32 scans per sample. The range of 4000-3656 cm-1, 2494-1819 cm-1 and <650 cm-1 was discarded due to distortion by CO2 and the ATR crystal (ZnSe). Microscopy of the sorbent surface was performed with a top-light microscope (Nikon SMZ645, G-AL 1.5x, Tokyo, Japan), digitized (SC50, Olympus, Tokyo, Japan) and are available in the supplements. The image resolution allowed for the observation of the macro surface structure in the range of 50 μm. The micro pore size distribution of sorbent materials was estimated using the BET surface area [28] data supplied by Eurofins Umwelt Ost GmbH (Appendix Figure A.6) and evaluated according to Dollimore and Heal (2007) and Viswanathan and Sastri (1967) [29,30].

### 2.2 sorption kinetics

The sorption kinetics with particular interest in the equilibration time and sorption capacity were determined first. The results were used together with the chemical/physical characterisation to select the two most suitable biochars for the subsequent isotherm experiments. The methodology was similar to the sorption experiments described below, but was performed with only three replicates from each sorbent as well as sampling and sample analysis on a daily basis. Sorption rate constants were calculated using the Lagergren first-order reaction kinetic [31].

(1) $$\begin{array}{c} \ln\left(q_{e}-q_{t}\right)=\ln\left(q_{e}\right)-k_{sorption}*t \end{array}$$

q_e_ and q_t_ are the adsorption capacities at equilibrium and at time t. k_sorption_ (min^−1^) is the first-order reaction rate constant. Equilibrium was defined as a change in the aqueous concentration of less than 5 % within 24 h.

### 2.3 Sorption experiments

The sorption isotherms were determined for the selected biochars (wood chip biochar pyrolysed at 550 °C (W550) and apricot kernel biochar pyrolysed at 750 °C (A750) and the AC for PCE, TCE, cDCE, VC and ethene in multi-solute and single-solute experiments. While single-solute experiments were carried out for ethene using a stock solution made from acetone, multi-solute experiments were carried out using a methanol multi-component stock of PCE, TCE and cDCE. VC was handled separately using a methanol stock solution. For sorption isotherms, 200.0 mg biochar or 25.0 mg AC was mixed with Milli-Q® water in 60 ml glass vials and sealed with Mininert® caps. Sorption isotherms were determined based on 8 single points per sorbent and three controls (aqueous phase only). Biochars were shaken in advance for 24 h to ensure wetted sorbent particles. Aliquots of the methanol stock solutions were added to the bottles leaving a final headspace of <0.5 mL. Aliquots of the spiked stock solutions were ≤0.6 mL to avoid co-solvent sorption effects at co-solvents mole fractions of >0.005 [32]. For ethene, 1 g of sorbent material was mixed with Milli-Q® water in 120 ml serum bottles and sealed with PTFE-lined grey butyl rubber septa and aluminium crimp-caps. GC-C/IRMS measurements were also used for compound quantification (measurement uncertainty of 30, 30, 50, 50 and 110 nmol L^−1^ for PCE, TCE, cDCE, VC and ethene, respectively). ETs were monitored using samples from the aqueous phase taken subsequently after preparation and after an equilibration period of seven days. Sorption experiments of CEs were repeated by altering the compound composition of the multi-component stock (PCE only; PCE, TCE and cDCE; PCE, TCE, cDCE and VC). Bottles were stored on an overhead shaker at 6 rpm in the dark and at room temperature (25 °C).

### 2.4 Compound-specific-stable-isotope analysis (CSIA)

The stable carbon isotope ratio of ETs was determined according to Leitner et al. (2017) [33]. Shortly, liquid samples were concentrated by a purge and trap autosampler, single compounds were separated and measured by gas chromatography coupled to isotope ratio mass spectrometry (purge and trap GC-C/IRMS). The stable carbon isotope ratios are given in the δ-notation in parts per thousand (‰) and were referenced against the Vienna Pee Dee Belemnite (VPDB) scale.

(2) $$\begin{array}{c} \delta^{13}C=\frac{R_{P}}{R_{Std}}-1\text{,} \end{array}$$

R is the ratio of the abundance of ^13^C/^12^C of a sample (P) and a measurement standard (Std – working gas) [34]. The δ^13^C vs VPDB values of PCE, TCE and cDCE were determined using the reference materials USGS87, NBS22 and IAEA–CH–3 [35,36]. For this purpose, one μl of pure PCE, TCE and cDCE respectively, as well as the international reference materials were filled into press-tight tin cups and analysed by elemental analysis coupled to isotope ratio mass spectrometry. (EA-IRMS, Thermo Fisher Scientific, Bremen, Germany. Assigned δ^13^C values of PCE, TCE and cDCE were −27.51 ± 0.13‰ (n = 5), −29.81 ± 0.08‰ (n = 3) and −25.94 ± 0.02‰ (n = 5) respectively. Referencing of the residual compounds VC and ethene was done when measured relative to PCE, TCE and cDCE analysed by purge and trap GC-C/IRMS using a multi-component calibration standard. Calibration standards of ETs were prepared in aqueous solutions and measured δ^13^C values (mean ± 1σ) for PCE, TCE, cDCE, VC, and ethene were −27.5‰ ± 0.3 ‰ (n = 39), −29.6‰ ± 0.2‰ (n = 53), −25.9‰ ± 0.3 ‰ (n = 59), −28.3‰ ± 0.1 ‰ (n = 11), and −29.1‰ ± 0.1‰ (n = 9), respectively, over the course of the sorption experiments.

### 2.5 sorption equilibrium

The sorption isotherms were calculated using the logarithmic form of the empirical Freundlich equation [37].

(3) $$\begin{array}{c} \log\left(q_{e}\right)=\log\left(K_{f}\right)+\frac{1}{n}*\log\left(c_{e}\right) \end{array}$$

The adsorbate mass per mass adsorbent is represented by q_e_ (mg g^−1^) and the adsorbate concentration in the aqueous phase by c_e_ (mg L^−1^), both at equilibrium and correlated by the linear regression constants K_f_ (mg ^1-1/n^ kg^−1^ L^1/n^) and 1/n (−).

(4) $$\begin{array}{c} K_{u}=K_{f}/C_{e}^{\frac{n-1}{n}} \end{array}$$

The unit-unifying factor K_u_ (L kg^−1^), according to Chen et al. (1999) [38], was used to compare the sorption capacities of the charcoals and for the chemicals and implied calculation of Freundlich isotherms calculated using molar units.

The isotope effect on sorption was calculated as the isotopic fractionation factor [34] of the sorption equilibrium (ε in ‰) [39], according to.

(5) $$\begin{array}{c} \varepsilon =\frac{\delta^{13}C_{s}*10^{-3}+1}{\delta^{13}C_{l}*10^{-3}+1}-1 \end{array}$$

δ^13^C corresponds to the stable carbon isotope ratio (in ‰) of the ETs in the solid (δ^13^C_s_) and aqueous liquid phase (δ^13^C_l_) at equilibrium, calculated for a closed system approach [19]. The carbon isotope ratio of the sorbed mass fraction (δ^13^C_s_ in‰) was calculated according to a mass balance equation, using the δ value at t = 0 (δ^13^C_0_) and t = equilibrium (δ^13^C_e_) determined by sampling of the aqueous phase and assuming a negligible loss of ETs to the gas phase or due to septum leakage.

(6) $$\begin{array}{c} \delta^{13}C_{s}=\frac{\delta^{13}C_{0}*c_{0}-\delta^{13}C_{e}*c_{e}}{c_{0}-c_{e}} \end{array}$$

The carbon isotope fractionation factors were calculated after data set cleaning using the isotopic difference [34] obtained from the isotherm experiments. The carbon isotope difference of each sorbent and ET, indicated by the shift in δ^13^C_l_ values between t = 0 and at the equilibrium (Δδ^13^C in ‰), were filtered to the span of the median value ±1σ for Δδ^13^C values larger than twice the GC/IRMS measurement uncertainty of calibration standards. Residual datasets were checked to be normally distributed in order to perform an iterative outlier test [40] with a significance level (α) of <0.05, for data sets with n > 6. The carbon isotope fractionation factors (ε), calculated from the remaining data sets, were filtered within the median ± 2σ to obtain the final values for each sorbent and ET. The latter enabled to also process data sets with n < 6 in order to exclude extreme values and normalise the data sets.

## 3 Results and discussion

### 3.1 Sorbent properties

Elemental analysis of the feedstock materials showed similar carbon content (∼50 %w) and some variation in nitrogen content (0.16–1.48 %w). The biochar yield decreased with increasing pyrolysis temperature (350 °C, 550 °C, 750 °C) and was independent of the feedstock (e.g. 35 and 17% w/w for apricot kernel biochar (A) and 28 and 14% w/w for woodchip biochar (W)). Similarly, BET surface area, pH-value, carbon content, and Σ16-EPA-PAHs (polycyclic aromatic hydrocarbons) increased with pyrolysis temperature and showed slight variation between the feedstock materials (Table 1 and in appendix (A) in Table A2 summarising all biochar variants). The H/C, O/H and (O + N)/C molar ratios showed a decreasing trend with increasing pyrolysis temperature, which was similar to the results reported in Ahmad et al. [41] and Schimmelpfennig and Glaser [42] presenting results using a similar pyrolysis reactor. Apart from apricot kernel biochar, sorbents produced at 750 °C had a Σ16-EPA-PAH content of 26–48 mg kg^−1^ and were excluded from further experiments because of exceeding PAH thresholds of the EBC-guideline. In addition, Miscanthus (M) biochar was also discarded due to the brittleness of the particles, therefore unsuitable for filter applications.

**Table 1:** Overview of results of the chlorinated ethenes (PCE, TCE, cDCE, VC) and ethene sorbed to the biochar A750 and W550 and the activated carbon (AC). Chemical/physical properties (BET surface area in m^2^ g^−1^, bulk density in g cm^−3^, porosity (−), C in %wt, (O + N)/C, O/C and H/C ratio (−), pH, Σ16-EPA-PAHs content (mg kg^−1^)); sorption kinetic parameters (equilibration time (t_equi_ in hours), rate constant k_sorption_ in min^−1^, coefficient of determination R^2^)); sorption isotherm parameters (K_f_ in mg ^1-1/n^ kg^−1^ L^1/n^, 1/n (−), number of data points (n).

	AC				W550				A750			
**BET**	1100.0				264.0				362.0			
**ρ**_**bulk**_	0.37				0.14				0.30			
**porosity**	0.82				0.89				0.83			
**C**_**org**_	88.6				89.6				88.2			
**(O + N)/C**	0.02				0.05				0.04			
**O/C**	0.01				0.04				0.03			
**H/C**	0.03				0.38				0.15			
pH	9.1				8.3				8.9			
**Σ16-PAH**	<0.1				5.6				0.7			
	**t**_**equi**_	**k**_**sorption**_	**R**^**2**^		**t**_**equi**_	**k**_**sorption**_	**R**^**2**^		**t**_**equi**_	**k**_**sorption**_	**R**^**2**^	
**PCE**	20				92	−5.0E-04	0.99		80	−6.0E-04	0.98	
**TCE**	20				92	−5.0E-04	0.99		140	−5.0E-04	1.00	
**cDCE**	20				44				153	−5.0E-04	1.00	
**VC**	20				20				153	−3.0E-04	0.99	
**Ethene**	149	−5.0E-04	0.56		29				168	−2.0E-04	0.78	
	**K**_**f**_	**1/n**	**R**^**2**^	**n**	**K**_**f**_	**1/n**	**R**^**2**^	**n**	**K**_**f**_	**1/n**	**R**^**2**^	**n**
**PCE**	189.7	0.49	0.95	20	7.4	0.40	0.92	23	1.3	0.72	0.94	7
**TCE**	45.3	0.44	0.97	20	6.7	0.47	0.96	23	1.5	0.69	0.91	7
**cDCE**	8.5	0.47	0.93	18	6.9	0.60	0.96	22	0.9	0.70	0.95	7
**vc**	3.4	0.85	0.95	8	3.3	0.63	0.97	14	0.8	0.75	0.94	7
**Ethene**	0.1	0.74	0.96	7	0.2	0.67	0.94	8	0.1	0.71	0.93	8
**%mass sorbed**												
**PCE**	98–99.93				52–99.37				57–84.27			
**TCE**	85–99.91				67–99.41				67–90.67			
**cDCE**	42–99.19				83–99.26				63–87.01			
**VC**	49–93.28				84–96.17				53–83.30			
**Ethene**	13–59.54				49–76.92				40–53.76

**Table 2:** Overview of results of the chlorinated ethenes (PCE, TCE, cDCE, VC) and ethene sorbed to the biochar A750 and W550 and the activated carbon (AC). Carbon isotope fractionation characteristics of AC, A750, W550 and the solid-free controls (range (Δδ^13^C_min_ – Δδ^13^C_max_) of carbon isotope differences from t = 0 to t = equilibrium (Δδ^13^C) in ‰ with negative values for the enrichment in^13^C-isotopologues in the aqueous phase, number of data points (n), %mass sorbed in the isotherm flasks (min – max), carbon isotope fractionation factor (ε) in ‰ (median ± two standard deviations (2σ) in ‰), lowest equilibrium concentration in the aqueous phase (c_a,min_) in μmol L^−1^).

	AC			W550			A750			control		
	Δδ^13^C		n	Δδ^13^C		n	Δδ^13^C		n	Δδ^13^C		n
**PCE**	−0.7–−1.8		5	−0.4–−1.3		10	0.1–−0.6		6	0.0–−0.5		8
**TCE**	−0.4–−0.9		4	−0.5–−0.9		7	0.0–0.2		5	0.0–−0.3		11
**cDCE**	−0.3–−0.5		5	−0.7–−1.6		12	0.0–−0.6		5	0.1–−0.3		6
**VC**	−0.1–−0.3		5	−0.8–−1.1		7	−0.5–−0.9		5	0.0–−0.3		3
**Ethene**	0.0–−0.8		7	−0.4–−1.1		7	−1.8–−2.4		6	0.0–0.2		3
**%mass sorbed**												
**PCE**	98.9–99.9			79.1–99.4			57.5–84.3			−17.0 – 12.8		
**TCE**	85.9–98.9			70.6–98.7			67.4–90.7			−10.3 – 8.1		
**cDCE**	65.5–99.2			94.2–99.3			62.7–87.0			−10.8 – 12.3		
**VC**	48.6–68.0			84.2–95.0			53.0–75.0			−18.2 – 17.0		
**Ethene**	13.0–59.5			49.0–76.9			40.4–53.8			−2.9 – 2.4		
	**ε**	**±2σ**	**c**_**a,min**_	**ε**	**±2σ**	**c**_**a,min**_	**ε**	**±2σ**	**c**_**a,min**_			
**PCE**	−1.5	0.4	0.03	−1.0	0.3	0.12	−0.5	0.4	0.24			
**TCE**	−0.7	0.3	0.57	−0.7	0.2	0.43	0.1	0.2	0.08			
**cDCE**	−0.5	0.2	0.05	−1.3	0.3	0.04	−0.7	0.4	0.06			
**VC**	−0.4	0.2	1.16	−1.1	0.1	0.48	−1.2	0.3	0.51			
**Ethene**	−1.0	0.4	1.18	−1.1	0.3	1.43	−4.4	0.6	2.86			

The sorption kinetic experiment, in alignment with chemical and physical EBC parameter threshold values identified the two most suitable biochars (W550, A750). A750 and W550 met the quality limits for EBC-Agro and EBC-AgroOrganic certification classes and compiled with the Σ16-EPA-PAH threshold values of 6 mg kg-1 [26]. The further pursued char variants W550, A750 and AC showed similar organic carbon content, but differences in BET surface area, aromaticity and degree of carbonization (H/C ratio), hydrophobicity (O/C ratio) and polarity ((O + N)/C ratio), as presented in Table 1. The biochars W550 and A750 showed a lower aromaticity, degree of carbonization, hydrophobicity and BET surface area and higher polarity when compared to AC.

### 3.2 Sorption characteristics

Sorption kinetics of ETs were described using the Lagergren pseudo-first order equation (Table 1), similar to previous studies dealing with charcoal sorption kinetics for aqueous/solid phase system experiments by Tseng et al. (2010) [43]. For an equilibrium within 48 h, the number of samples analysed within was too little to calculate the parameters of the sorption kinetic. Most important, sorption equilibrium with AC was reached within 20 h for CEs and within 149 h for ethene. W550 showed variable equilibration times from 20 to 92 h, which decreased with molecule size and water solubility (PCE > TCE > cDCE > VC). A750 showed the highest equilibration time for all ETs of up to 7 days, which determined the overall duration of the sorption isotherm experiments.

**Fig. 1:**
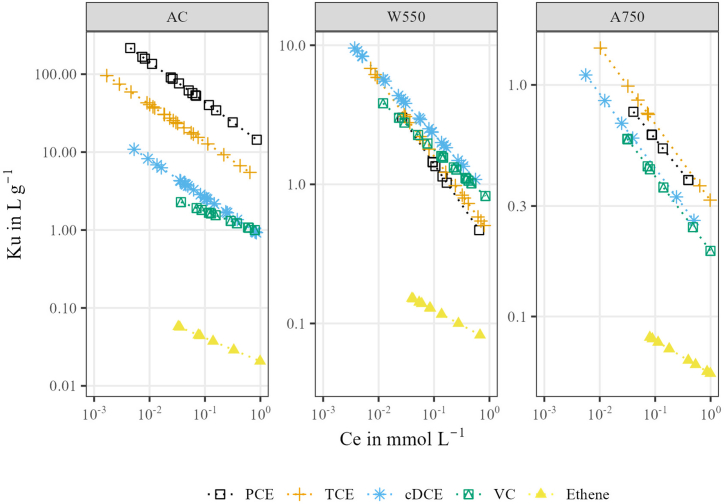
Overview of the sorption capacities of the coals for PCE, TCE, cDCE, VC and ethene, determined from Freundlich isotherms calculated from molar units and converted to the unit-uniform parameter Ku.

The sorption equilibrium was described best using the empirical Freundlich model when compared to the Langmuir model (higher coefficients of determination (R^2^), Table 1 and A1), which was similar to the results of previous studies [44,45]. The formation of multiple layers of CEs onto the sorbent surface was assumed when considering the CE's opposite binding affinity and because the sorption equilibrium was better described using the Freundlich model. The proportion of sorbed mass was from 13 % to >99.9 % which is beneficial for covering the non-linear part (elevated values of c_e_) of the Freundlich isotherm and to investigate isotope effects over a wide range of equilibrium concentrations. The magnitudes of the K_f_ and K_u_ values (Table 1 and Fig. 1) indicated that AC was the strongest sorbent for the more hydrophobic PCE and TCE. The sorption capacity of AC was in general attributed to the high specific surface area and sorption due to pore filling processes [46] since the carbon content and porosity were similar to that of the biochars. Sorption of PCE and TCE on the biochars, which showed a higher polarity compared to AC, was also associated with pore filling processes as well as partitioning. TCE was more strongly sorbed to biochars than PCE, because TCE has a higher aqueous solubility due to its higher polarizability (9.75 Å³ [47], in comparison to zero for PCE.

The most polar ETs, cDCE and VC (1.13 and 1.22 Debye [48]), were most sorbed by W550, reasoned by the comparatively higher O/C ratio suspecting sorption via the formation of hydrogen bonds. FTIR spectroscopy revealed that only W550 had hydrogen bonding potential, as only W550 showed peaks at wavenumbers 875, 820, 760 cm^−1^ (Figure A1) representing aromatic C–H bonds [49] and at 1600 cm^−1^ indicating aromatic C=O and C=C double bonds originated from lignin [50].

Microscopic examination of the sorbent particles revealed a smooth and homogeneous surface structure of large macropores (pores >50 μm) of AC and W550, with occasional cracks due to wood fibre lamellae for the latter (Figure A.4, Figure A.3). In general, pores were classified according to Sing (1985) as recommended by Thommes et al. (2015) [51,52]. In contrast, A750 (Figure A.2) had a heterogeneous surface morphology with deep macropores up to 200 μm in diameter, which may have resulted from evaporation of fatty acids deposits of the apricot kernels [53,54]. Furthermore, A750 exhibited weak wettability compared to AC, which could be attributed to dead-end pores, at similar bulk density. The similarly low wettability of W550 was rather explained by its low bulk density (0.14 g cm^−3^) and the fact of entrapped gas. Observations showed, that a high proportion of biochar particles began to sink only after several hours of continuous shaking. More difficult displacement of the gas phase present in the assumed dead end pores of A750 may also have been the reason why the sorption capacity of A750, despite its high specific surface area, was lower than that of W550 [55]. In addition, sorption capacity of A750 did not change among CEs, which in terms of enthalpy- and entropy-driven sorption processes would favour either cDCE due to its dipole moment or PCE due to its hydrophobicity, neither of which was the case. The enthalpy of adsorption is less negative with increasing sorbent loading [56], which is why the relatively high equilibration times of A750 compared to AC and W550 also suggested differences in sorbent surface homogeneity and pore accessibility.

The BET surface area data would give A750 clear preference over W550 when sorption mechanisms were the same (Table 1). However, in addition to surface area and feedstock specific chemical nature [57], the micropore size distribution has also to be taken into account. Jeong et al. (2016) [58] had shown that the pore size distribution of biochar can shift in favour of micropores (<2 nm) when pyrolysis temperatures were increased to 750 °C. According to Zhao et al. (2019) [59], the average pore diameter of biochar can decrease by 30–210 % to 2.5–3.4 nm by increasing the pyrolysis temperature from 500 to 700 °C. Schreiter et al. (2018) [4] concluded that pore size distributions were found in the range of 1 nm even for biochar produced at 450 °C. In order to obtain pore-size-exclusion mechanisms for the sorption of CEs, A750 must consist mostly of micropores with diameters less than 1.8 times the largest atomic-atom distance of CE molecules [23,60]. Surface approximations based solely on BET isotherms with nitrogen limit the interpretation and should only be used for a qualitative description of the results, especially when the material analysed contains molecular-sized pores [61].

ETs (ethene, VC, cDCE, TCE, PCE) have a spherical diameter of 0,39–0,69 nm [48], which can cause a pore size exclusion effect of micropores at and below the lower end of the BET nitrogen isotherm [62]. The externally provided BET isotherm data of nitrogen was used to estimate the pore size distribution at the relative pressure of 0.007–0.3 (P/P_0_) equivalent to a pore width of 1.3–3 nm. Calculation of the pore size distribution according to Dollimore and Heal (1964) [29] revealed a similar pattern in the pore size distribution of W550, A750 and AC at pore widths of 1.3–2.8 nm (Figure A.5), but different in magnitude when scaled to the respective surface area per pore width. The comparison of the BET surface areas of 1.3–3 nm showed similar proportions for W550 and A750 (56 and 60 m^2^ g-1 respectively and 367 m^2^ g-1 for AC (Table A.1)), since the difference in the BET surface area was due to pores <1.3 nm, which are rather inaccessible for the molecules of PCE, TCE and cDCE. According to Mukherjee et al. (2011) [54] increasing the pyrolysis temperature from 400 to 650 °C lead to the release of volatile components thereby mostly increasing the surface area represented by pores <1.5 nm. In view of the higher sorption of the polar and temporarily polarizable VC on W550 and similar sorption of poorly ionisable ethene, it was concluded that the difference in sorption capacity lies on the one hand in the high polarity and wealth of functional groups in W550 compared to A750. Furthermore, the excess of pores <1.3 nm in A750 compared to W550 and this outstanding proportion in AC for the sorption of ethene should not play a decisive role, since ethene was adsorbed equally little by all three sorbents.

### 3.3 Isotope effects at the sorption equilibrium

Prior to raw data processing, the data sets were checked for kinetic isotope fractionation according to the Rayleigh equation [63] and for the impact of sorbed mass fraction (13–99.93%) on the isotopic difference (Figure A.10), both of which were then discarded due to minor correlation. The influence of the evaluation and processing of the raw data obtained from the isotherm experiments led to a reduction in the standard deviations (σ) of Δδ^13^C and ε values, while their median values remained constant in the course of the individual evaluation steps as indicator of the high data quality (Figure A.7 and A.8).

**Fig. 2:**
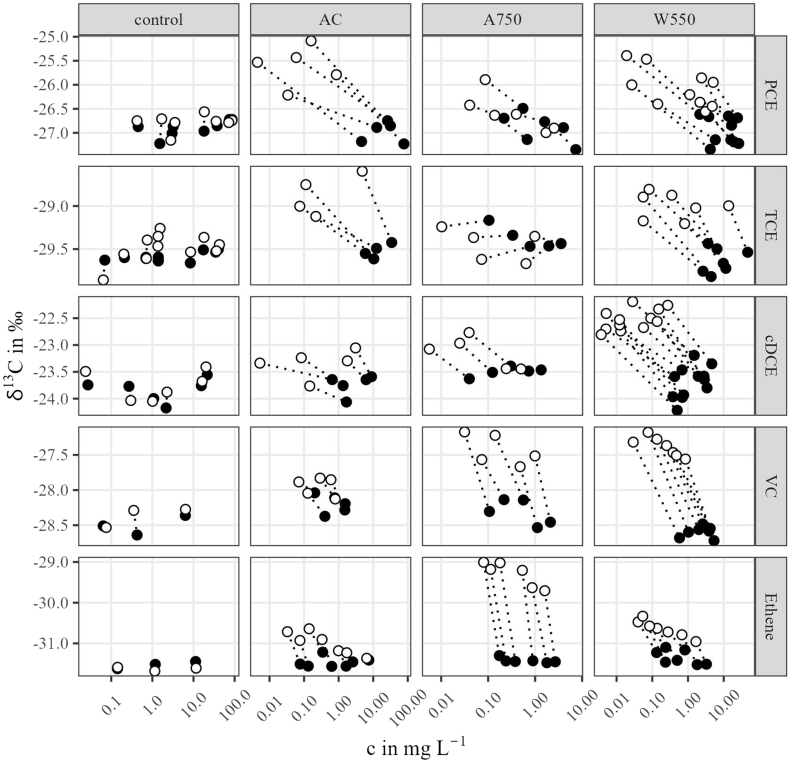
Summary of shifts in δ^13^C values (in ‰) and the respective concentration (c in mg L^−1^) in the aqueous phase of the isotherm experiments (final data set after processing) covering PCE, TCE, cDCE, VC and ethene exposed to the sorbents AC, W550, A750 and a sorbent-free aqueous control. Filled and open symbols depict concentrations at t = 0 and at the equilibrium, respectively.

The CEs were added from a multi-solute stock in order to prevent uneven variation in their contents compared to single compound addition. This also involved the identification of leaks, handling errors and quantification errors. There were still fluctuations in the recovery of the added CEs in the controls (Table 2). The variation in mass recovery probably resulted from the fact that the controls took longer to mix the added CE due to the absence of a solid, thus affecting sampling at t = 0. However, the fluctuation in the contents did not show any effect on the isotope ratio values, as these were weaker compared to fractionation in the presence of the sorbents. In general, isotope fractionation was also assumed not to be affected by vaporization, because it would have caused ^12^C enrichment in the aqueous phase [12], which was not observed in the controls, nor for the sorbents (Fig. 2). It was also ruled out that the AC, W550 and A750 isotherm experiments were affected by level fluctuations of the same magnitude as the controls, since such would have been recognized as outliers and would not have been taken into account in either the isotherm data analysis or the isotope effects assessment.

The analytic scatter of the δ^13^C values at t = 0 per ET and over all sorbent flasks ranged from 0.13 to 0.25‰. Controls indicated a maximum change in δ13C values from the initial to the final sampling (Δ^δ13^C_max_) of 0.2 to −0.5‰ (Ethene < VC < TCE < cDCE < PCE) with positive/negative values indicating 13C depletion/enrichment (Table 2), mostly in favour of 13C enrichment in the aqueous phase. For sorbents, shifts in δ^13^C values were more pronounced for most sorbent/ET pairs than in the controls, with an isotope difference in the aqueous phase of up to −1.8 ‰, −0.9 ‰, −1.6 ‰, −1.1 ‰ and −2.4 ‰ (Δδ^13^C_max_) for PCE, TCE, cDCE, VC and ethene, respectively. Apart from the magnitude of the isotopic difference, the range was mostly more pronounced than in the controls and showed evidence of a dependence on the combination of sorbent and ET (Table 1), but not a significant trend on the adsorbed mass fraction (Fig. 2). The magnitude of the δ^13^C shift of PCE and TCE was largest on AC, for cDCE on W550 and for VC and Ethene in combination with A750 (Table 2, Figure A.9).

Carbon isotope fractionation factors (ε in ‰) for the sorption of ETs onto AC, W550 and A750 were found to reflect enrichment of ^12^C-bearing isotopologues on the sorbent at an extent of −0.4 to −4.4 ‰ within 2σ of ±0.1 to ± 0.6 ‰. Only the sorption of TCE onto A750 was found to show less pronounced isotope fractionation, while ethene showed the overall largest fractionation when sorbed to A750.

Since we could not find any clear indications of an isotope effect of a kinetic nature either in the kinetics experiments or the isotherm experiments, the observed isotopic effects in the sorption equilibrium were attributed to thermodynamics (i.e. the fractionation factors cannot only be attributed to sorption and diffusion processes may also have been involved). The extent of the fractionation by diffusion processes is much more pronounced in the case of dissolved gases than in the case of liquids [64,65]. Ethene showed a comparatively high carbon isotope fractionation during sorption on the biochar A750 in comparison to the other sorbents and also in comparison to the CE. The surface structure of A750 showed a high proportion of nano pores but also deep macropores compared to the other two sorbents. With regard to the macropores, the poor water wettability and the comparatively long equilibration time of ethene, it seems reasonable to assume that the observed fractionation was also influenced by diffusion processes in partly gas-filled macropores.

The major difference in the methodological approach to previous studies [e.g. 4,16,21], which concluded on negligible isotopic fractionation for CEs within GC/IRMS measurement uncertainties of ±0.5‰, was the use of coarse sorbent particles of 0.6–2.0 mm instead of powdered particles. In general, capacity for ET sorption had been shown to largely depend on the sorbent properties, like pore size distribution as well as surface chemistry [e.g. 23]. Therefore, it was assumed that using coarser-grained sorbent material could better reveal isotope effects during sorption equilibrium. Of course, the use of coarse-grained particles raises the issue of diffusion. The extent to which sorption and diffusion processes, both of which lead to the accumulation of sorbate molecules on the surfaces of the pore matrix of sorption particles, can/should be separated from each other is questionable and depends on the research question. In our opinion, the isotope effects derive from the wider sorption process, since the sorption capacity remains decoupled from diffusion, while the latter acts inside the pore spaces of the sorbent particle.

With the exception of TCE adsorbed to A750, the results consistently demonstrated an accumulation of ^13^C isotopologues in the aqueous phase. This indicated that ^12^C isotopologues remained more easily sorbed on the surface, permanently, which qualitatively reflected the results of Höhener and Yu (2012), Imfeld et al. (2014) and Wanner et al. (2017) [20,22,66]. A comparison of the mass sorption and isotope effects showed that for AC and W550 those ETs that had the highest sorption capacity also showed the largest isotope fractionation (Table 2). This lead to the assumption that fractionation processes are related to the binding forces that prevail during sorption. As discussed previously for sorption capacity, nano and micropore size distribution was also considered as a possible cause of isotopic fractionation. At low temperatures (<100 K) sorbents with a pore size distribution tailored to the adsorbate can be used to enrich deuterium over protium. This shows an isotope effect, with an enrichment of the heavy isotope on the sorbent in the range of one power of ten [14,67,68]. At elevated temperatures, such as at standard temperature and pressure (S.T.P.), these separation effects subside, but can still, according to the data presented, be expressed as an isotope effect for the liquid/solid phase equilibrium of chlorinated groundwater pollutants with a significant change in the ^13^C/^12^C ratio using GC/IRMS instrumentation. Despite the fact that heavier isotopologues are the smaller molecules with higher kinetic energies and lower activation energies for sorption and chemically similar surfaces, these can also be transiently more easily polarized, increasing interaction with polar solvent water molecules and thus freedom of movement in pore spaces.

## 4 Conclusions

The presented study dealt with the sorption capacities and isotope effects of ETs (chlorinated ethenes and ethene) in sorption equilibrium with biochar and activated carbon, which could be linked to the chemical surface properties and the particle pore size distribution. Isotope effects in the sorption equilibrium were observed and the apparent isotope fractionation factors were determined. The isotope effects in the sorption equilibrium were assumed to also arise from diffusion processes that are inseparable from sorption and that do not affect the sorption capacity in the equilibrium, but can influence the sorption kinetics on a temporal scale.

Microbial degradation of CE shows high variability in carbon isotope fractionation, ranging from insignificant isotope differences to fractionation factors (ε) of −18.9 ‰ [33,69]. As a consequence, higher threshold values should be considered or interpreted more conservatively for the identification and evaluation of pollutant-degrading processes due to shifts in the carbon isotope ratio of up to −2.4‰. Although we could not clarify the origins of the observed fractionation processes, we discussed potentially involved processes in order to provide starting points for subsequent work. As an example, the application of two-dimensional CSIA (e.g. chlorine [70]) could potentially provide further insight into the contribution of sorption and diffusion in pore systems of sorbent particles. The results presented thus make an important contribution to the differentiation from equilibration (sorption, diffusion) and transformation (degradation) processes of environmental pollutants.

## Data availability statement

Data will be made available on request.

## CRediT authorship contribution statement

**Simon Leitner:** Conceptualization, Data curation, Funding acquisition, Investigation, Methodology, Project administration, Supervision, Visualization, Writing – original draftWriting – original draft. **Fridjof Sobanski:** Data curation, Investigation, Resources. **Gerhard Soja:** Conceptualization, Investigation, Resources, Supervision, Writing – review & editingWriting – review & editing. **Katharina Keiblinger:** Conceptualization, Investigation, Resources, Supervision, Writing – review & editingWriting – review & editing. **Christine Stumpp:** Conceptualization, Funding acquisition, Project administration, Resources, Supervision, Writing – review & editingWriting – review & editing. **Andrea Watzinger:** Conceptualization, Formal analysis, Funding acquisition, Investigation, Project administration, Supervision, Writing – review & editingWriting – review & editing.

## Declaration of competing interest

The authors declare the following financial interests/personal relationships which may be considered as potential competing interests:Andrea Watzinger reports financial support was provided by Kommunalkredit Public Consulting GmbH. Andrea Watzinger reports equipment, drugs, or supplies was provided by Donau Carbon GmbH.
