# Supplementary material for: Carbon isotope effects in the sorption of chlorinated ethenes on biochar and activated carbon
Source: Heliyon. 2023 Oct 10;9(10):e20823. doi: 10.1016/j.heliyon.2023.e20823 (PMC10590956; doi:10.1016/j.heliyon.2023.e20823)
Supplement: Multimedia component 1 [file mmc1.docx]

Carbon isotope effects in the sorption of chlorinated ethenes on biochar

*Simon Leitner^a^, Fridjof Sobanski^a^, Gerhard Soja^c,d^, Katharina Keiblinger^a^, Christine Stumpp^b^ & Andrea Watzinger^a^*

^a^ University of Natural Resources and Life Sciences, Vienna, Institute of Soil Research, Konrad-Lorenz-Straße 24, 3430 Tulln, Austria

^b^ University of Natural Resources and Life Sciences, Vienna, Institute of Soil Physics and Rural Water Management, Muthgasse 18, 1190 Vienna, Austria

^c^ AIT Austrian Institute of Technology, Konrad-Lorenz-Straße 24, 3430 Tulln, Austria

^d^ University of Natural Resources and Life Sciences, Vienna, Institute of Chemical and Energy Engineering, Muthgasse 107, 1190 Vienna, Austria

**Corresponding Author: Simon Leitner, simon.leitner@boku.ac.at, +43 14765491177**


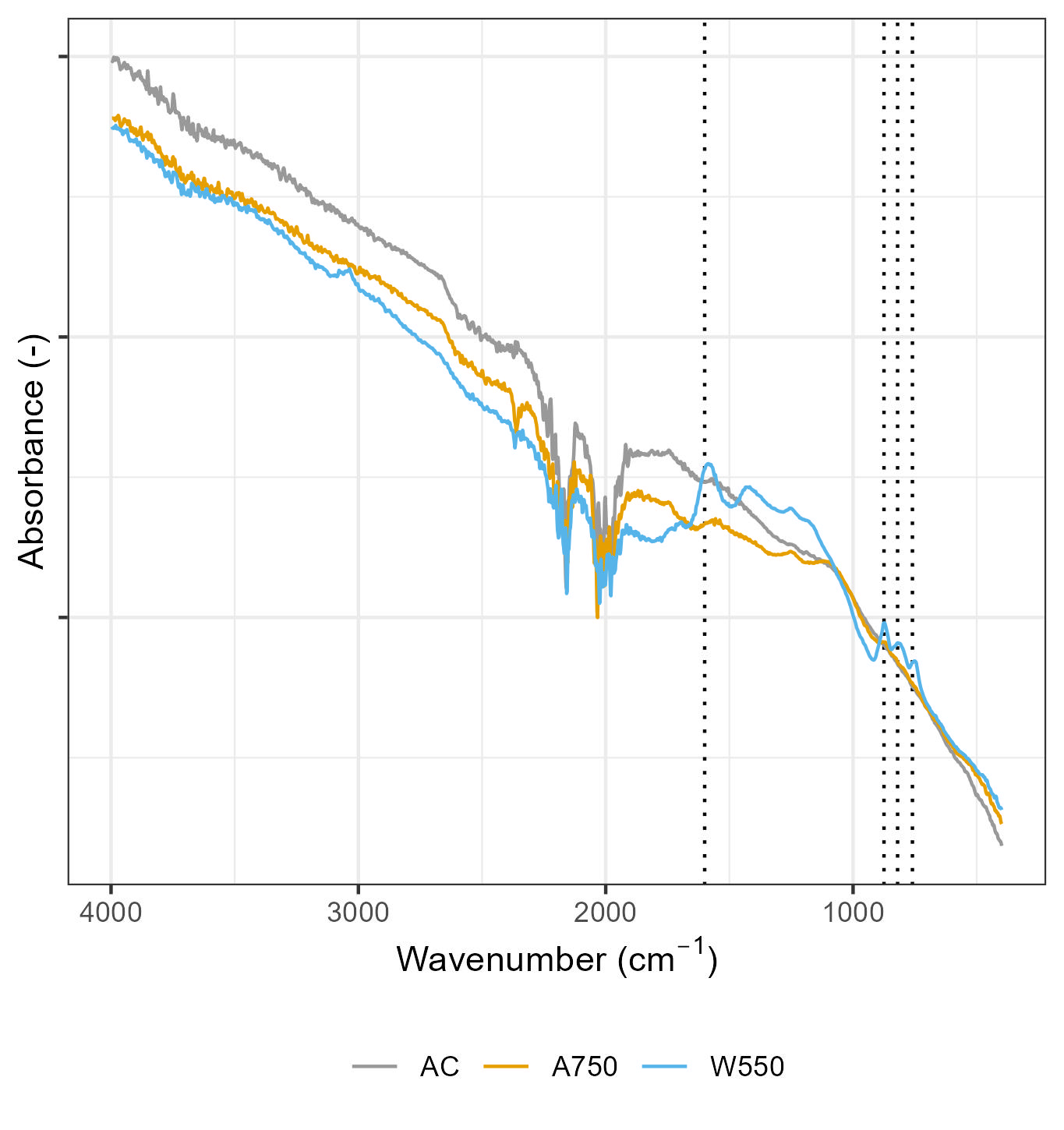


Figure A. 1: FTIR spectroscopy of the sorbents AC, A750 and W550 (activated carbon (AC), apricot kernel biochar (A) pyrolysed at 750°C, wood chip biochar (W) pyrolysed at 550°C). Dotted lines indicate the Wavenumber 1600, 875, 820 and 760 cm^-1^.


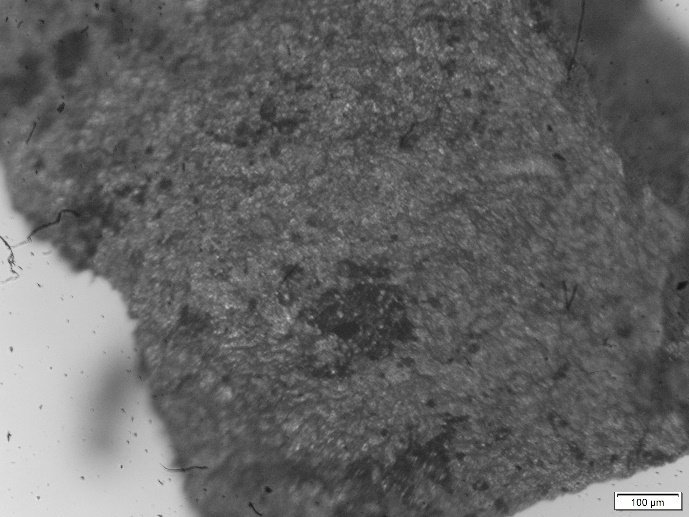

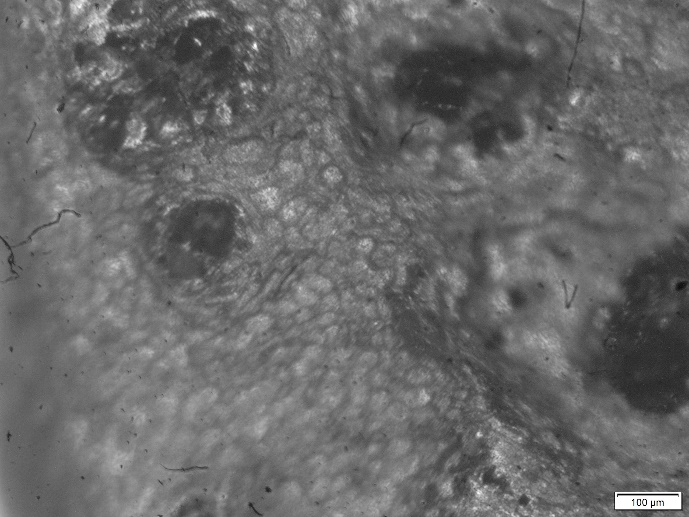

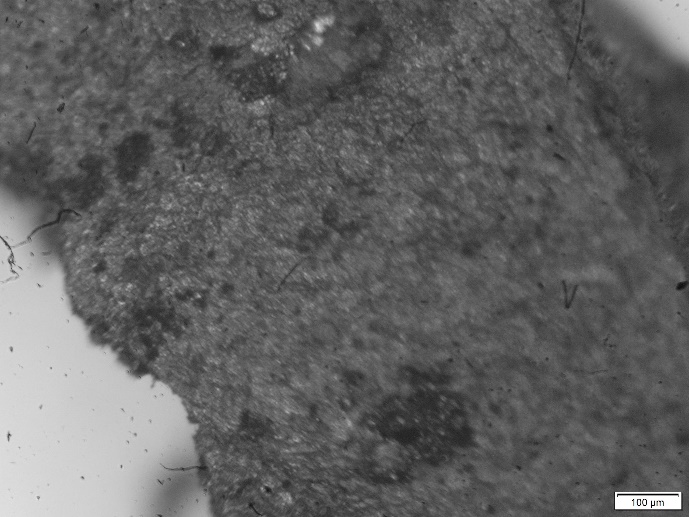


Figure A.2: Images of the surface of biochar A750.


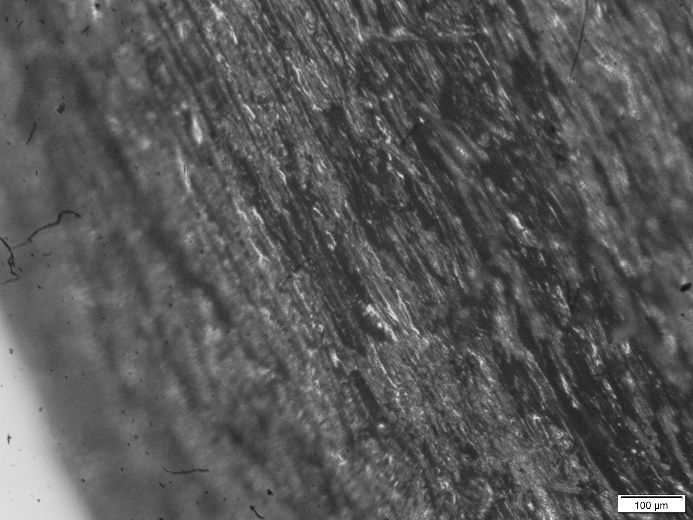

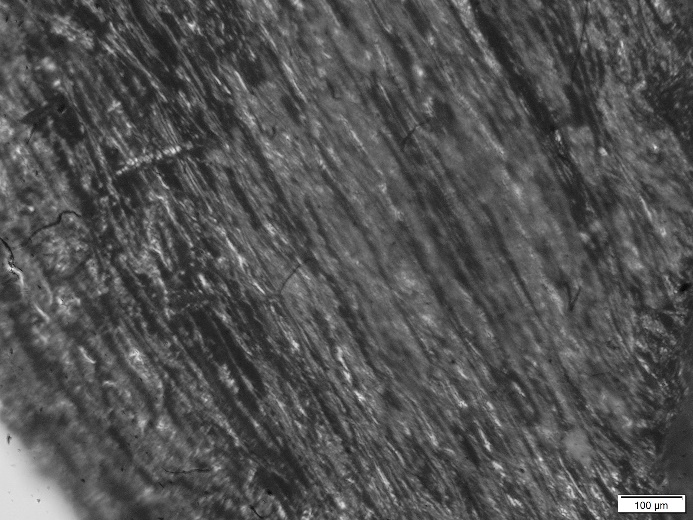

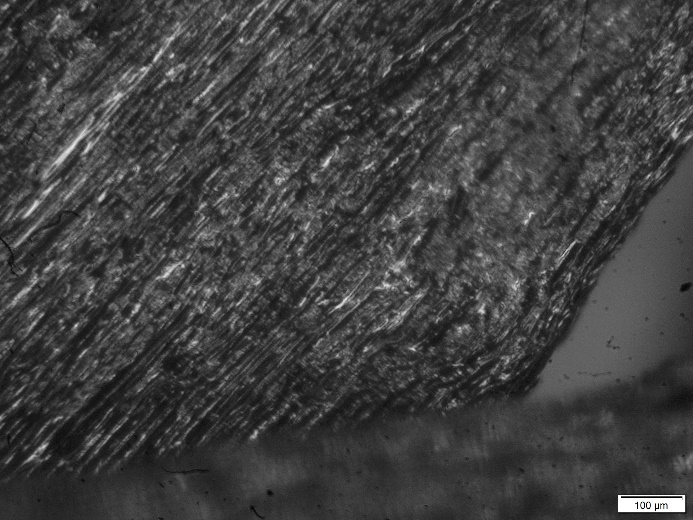


Figure A.3: Images of the surface of biochar W550.


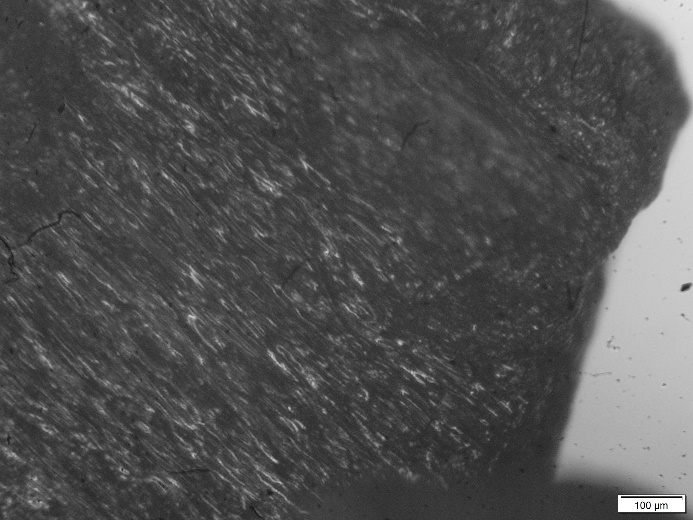

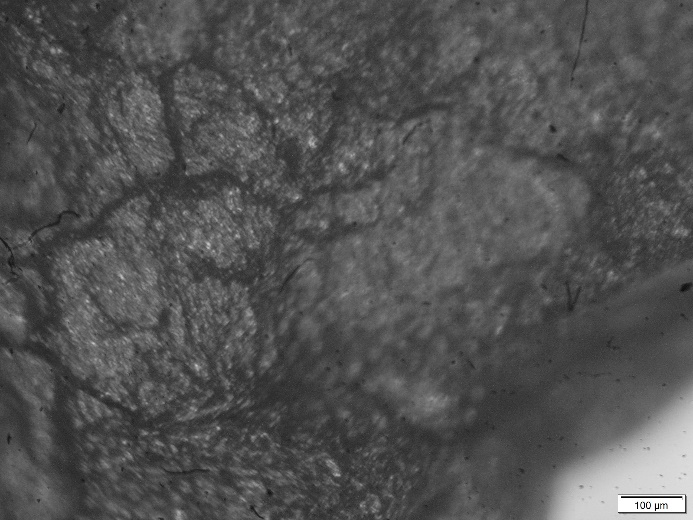


Figure A.4: Images of the surface of the activated charcoal (AC).


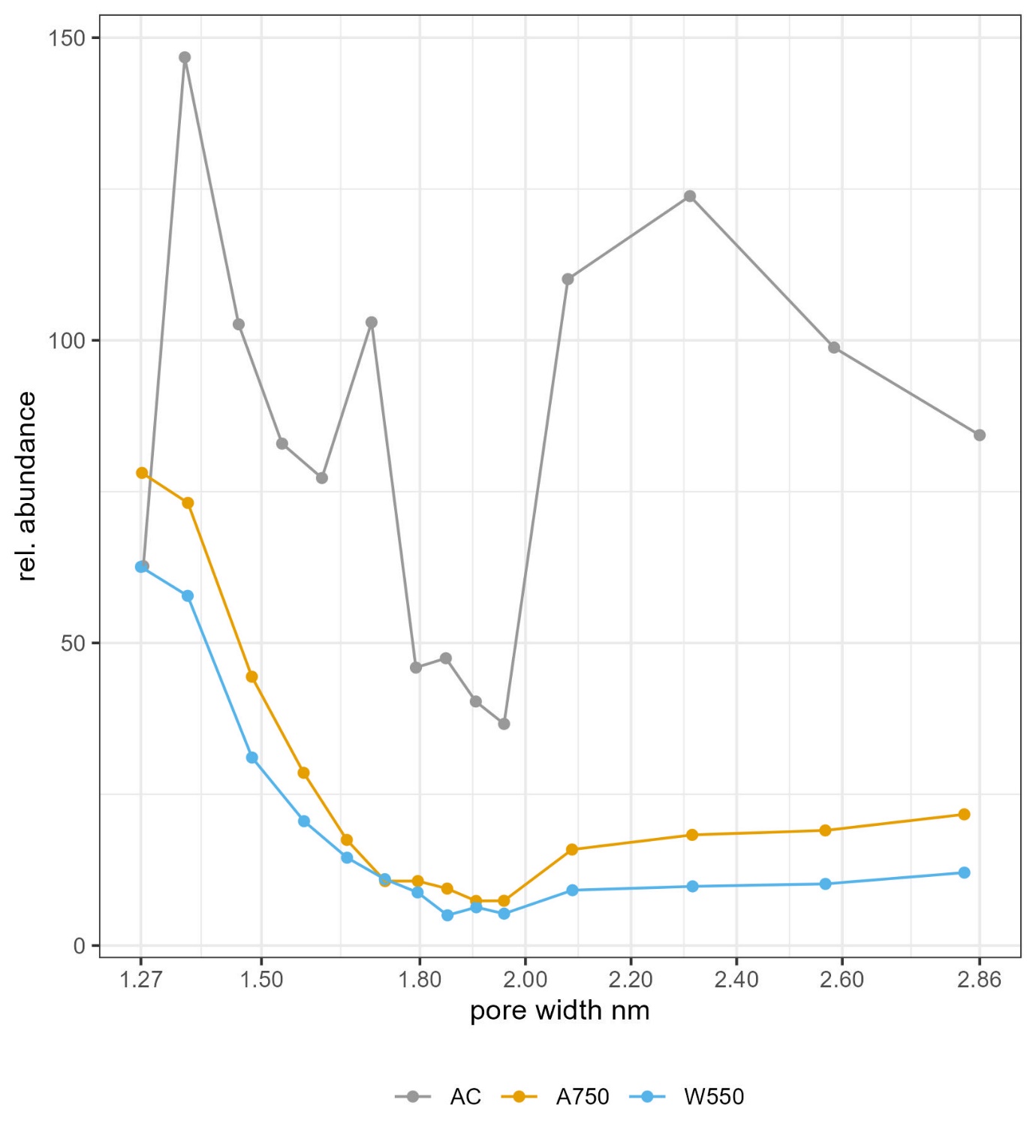


Figure A.5: Pore size distribution of the sorbents AC, A750 and W550 calculated from the BET surface area Data according to Dollimore and Heal (1964). The x-axis shows the pore width in nm, assuming cylindrical pores and the y-axis shows the respective share of the BET surface area (m² g^-1^) according to the ratio of adsorbed N_2_-filled pore volume (V_p_) calculated as relative abundance (dV_p_/sum(V_p_)*BET).


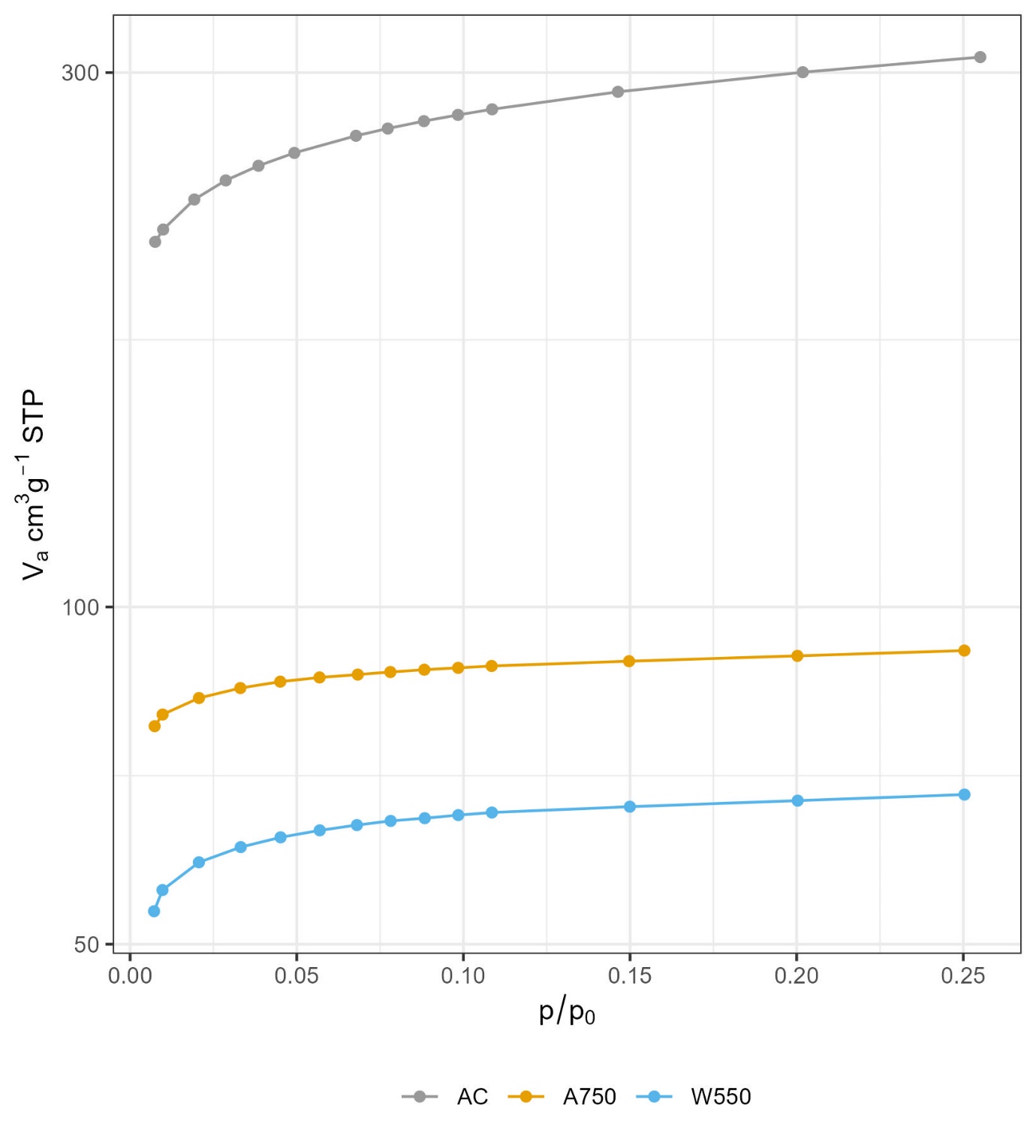


Figure A.6: BET-Isotherms of nitrogen adsorption for the sorbents AC, A750 and W550 plotted for the relative pressure increase (p/p0) to the adsorbed Volume of nitrogen in mL per g at standard pressure and temperature (STP).

Table A.1. Results of the BET surface Area data determined from Nitrogen adsorption with Vads as the Volume of adsorbed Nitrogen and p/p0 as the relative pressure with p0 indicated the saturation vapour pressure at 77.3 °K.

| **sorbent** | **N2 BET surface area** | **Vads at p.p0=max(p.p0)** | **Vads at p.p0=min(p.p0)** | **ratio Vads pore width <1.3nm** | **N2 BET at pore width <1.3nm** | **N2 BET at pore width 1.3-2.9 nm** |
| --- | --- | --- | --- | --- | --- | --- |
|  | **m² g^-1^** | **ml STP** | **ml STP** |  | **m² g^-1^** | **m² g^-1^** |
| AC | 1100.0 | 318 | 212 | 67% | 733 | 367 |
| A750 | 362.0 | 93 | 78 | 85% | 306 | 56 |
| W550 | 264.0 | 69 | 54 | 78% | 205 | 60 |

*Table A.2. Overview of sorbent chemical and physical properties of the produced biochars (Miscanthus (M), Sunflower seed shells (S), wood chips (W), apricot kernels (A)) and the activated carbon (AC).*

| **Material** | **Temp.** | **porosity** | **ρ_b_** | **BET** | **pH** | **Σ16-PAH** | **C** | **H** | **O** | **N** | **H/C** | **O/C** | **(O+N)/C** |
| --- | --- | --- | --- | --- | --- | --- | --- | --- | --- | --- | --- | --- | --- |
|  | °C |  | g cm-³ | m² g-1 |  | mg kg-1 | wt% | wt% | wt% | wt% |  |  |  |
| A | 350 | 0.76 | 0.34 | - | 7.3 | 0.1 | 73.8 | 4.8 | 16.1 | 1.83 | 0.8 | 0.16 | 0.19 |
| A | 550 | 0.78 | 0.3 | 178 | 8.1 | 2.1 | 82.9 | 2.8 | 8.0 | 1.63 | 0.4 | 0.07 | 0.09 |
| A | 750 | 0.83 | 0.26 | 363 | 8.9 | 0.7 | 88.5 | 1.1 | 3.0 | 1.47 | 0.2 | 0.03 | 0.04 |
| W | 350 | 0.88 | 0.14 | 5 | 8.9 | 0.7 | 76.5 | 3.9 | 17.9 | 0.22 | 0.6 | 0.18 | 0.18 |
| W | 550 | 0.89 | 0.14 | 264 | 8.3 | 5.6 | 89.8 | 2.8 | 5.1 | 0.55 | 0.4 | 0.04 | 0.05 |
| W | 650 | 0.89 | 0.21 | 331 | 9.0 | 3.5 | 82.6 | 0.9 | 4.1 | 0.69 | 0.1 | 0.04 | 0.05 |
| W | 750 | 0.92 | 0.13 | 424 | 9.3 | 32.4 | 94.7 | 1.1 | 1.2 | 0.77 | 0.1 | 0.01 | 0.02 |
| M | 550 | 0.93 | 0.11 | 216 | 9.2 | 4.8 | 84.6 | 2.3 | 4.5 | 0.54 | 0.3 | 0.04 | 0.05 |
| M | 750 | 0.95 | 0.09 | 453 | 9.4 | 26.2 | 84.3 | 0.9 | 3.4 | 0.75 | 0.1 | 0.03 | 0.04 |
| S | 350 | 0.82 | 0.36 | 8 | 10.0 | 7.4 | 76.3 | 3.4 | 11.7 | 1.51 | 0.5 | 0.12 | 0.13 |
| S | 550 | 0.94 | 0.1 | 83 | 9.1 | 2.0 | 83.0 | 2.1 | 4.7 | 1.21 | 0.3 | 0.04 | 0.06 |
| S | 650 | 0.86 | 0.24 | 154 | 9.5 | 2.0 | 73.4 | 0.8 | 4.8 | 3.1 | 0.1 | 0.05 | 0.09 |
| S | 750 | 0.95 | 0.09 | 362 | 10.9 | 48.4 | 83.1 | 0.9 | 4.1 | 1.17 | 0.1 | 0.04 | 0.05 |
| AC | - | 0.82 | 0.37 | 1100 | 9.1 | 0.0 | 88.6 | 0.2 | 1.4 | 0.79 | <0.1 | 0.01 | 0.02 |

The parameters of the Langmuir-Isotherm (q_max_ in l mg^-1^, K_l_ in mg g^-1^) were calculated using the linear regression of 1/c_e_ (equilibrium concentration in the liquid phase in mg l^-1^) vs. 1/q_e_ (equilibrium concentration in the solid phase in mg g^-1^) to solve the Langmuir equation.

$$q_{e}=\frac{q_{max}*K_{l}*c_{e}}{1+K_{l}*c_{e}}$$

Table A. 3. Results of the Langmuir isotherm parameters (Kl, qmax) as well as the number of data points (n) and the coefficient of determination (R²) for the adsorbates (PCE, TCE, cDCE, VC and ethane) and the sorbents AC, W550 and A750.

|  | **AC** | | | | **W550** | | | | **A750** | | | |
| --- | --- | --- | --- | --- | --- | --- | --- | --- | --- | --- | --- | --- |
|  | **K_l_** | **q_max_** | **R²** | **n** | **K_l_** | **q_max_** | **R²** | **n** | **K_l_** | **q_max_** | **R²** | **n** |
| **PCE** | 36.6 | 81.0 | 0.81 | 20 | 43. 0 | 24.7 | 0.40 | 23 | 0.9 | 11.8 | 0.98 | 7 |
| **TCE** | 33.2 | 19.0 | 0.95 | 20 | 45.0 | 24.2 | 0.87 | 23 | 45.2 | 42.0 | 0.97 | 7 |
| **cDCE** | 14.6 | 31.9 | 0.91 | 18 | 33.9 | 45.1 | 0.86 | 22 | 26.1 | 0.5 | 0.98 | 7 |
| **vc** | 44.7 | 32.9 | 0.71 | 8 | 45.0 | 45.1 | 0.98 | 14 | 45.0 | 0.7 | 0.74 | 7 |
| **Ethene** | 46.4 | 0.1 | 0.87 | 7 | 0.9 | 0.3 | 0.90 | 8 | 0.6 | 0.2 | 0.97 | 8 |


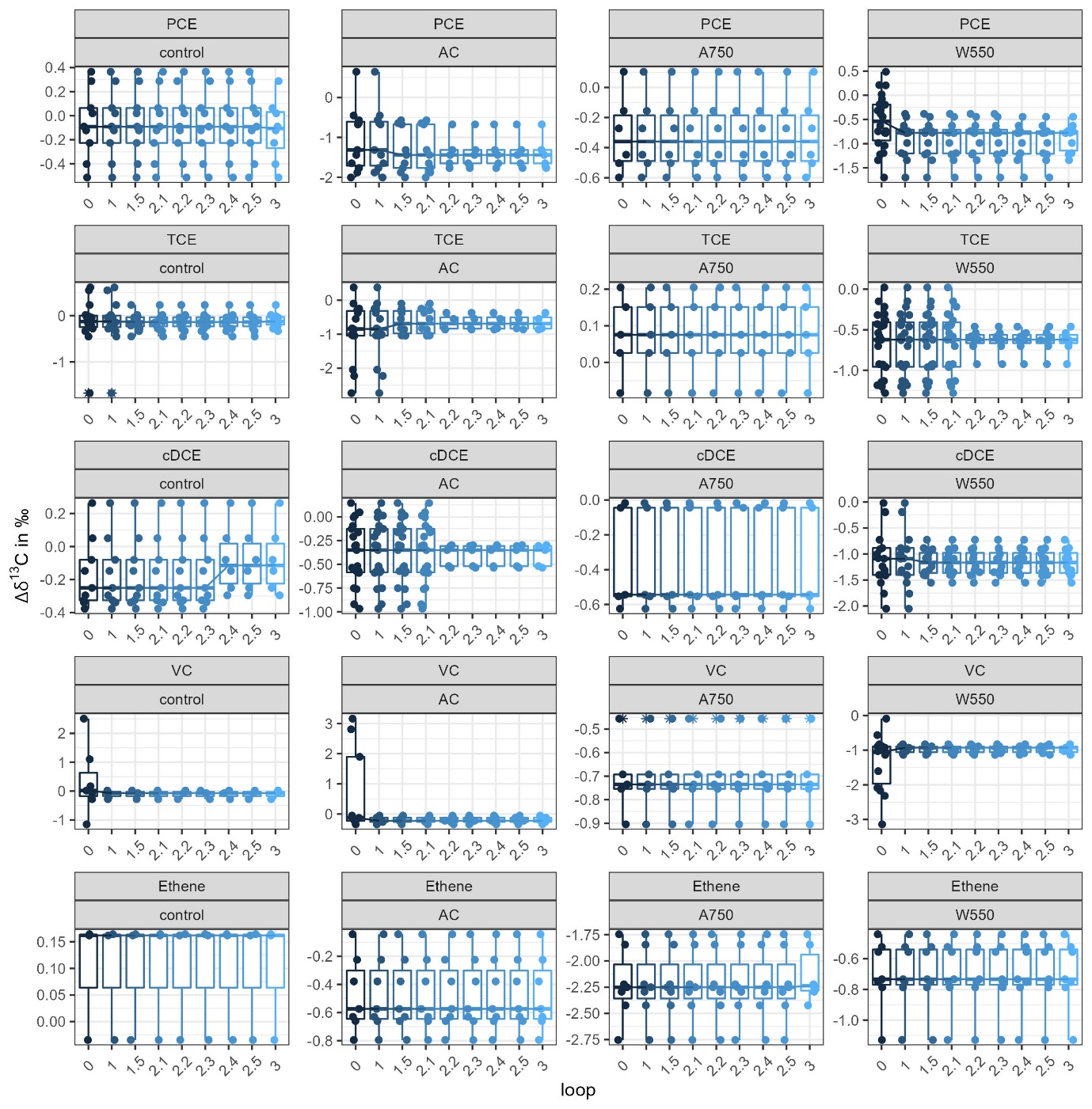


Figure A.7: Carbon isotope difference (∆δ^13^C in ‰ as shift in δ^13^C from t=0 to t=equilibrium) of ETs (PCE, TCE, cDCE, VC, ethene) at the sorption equilibrium with the charcoals AC, W550 and A750. The x-axis shows the continuous filtering of raw data sets (loop=0) to the final data sets (loop=3). Loops 0-3 represent the processing steps 1 (exclusion of isotherm experiments, which failed due to sampling and measurement issues), 1.5 (filtering of data sets within the median ± 1 σ), 2.1 – 2.5 (Grubbs outlier tests at a significance level α<0.05 and a minimum number of ∆δ^13^C above n=6 to check for 2 outliers on one side (2.1); one outlier on each side (2.2); single outlier for the maximum/minimum difference from the mean (2.3, 2.4) and after the iterative outlier tests (2.5)) and 3 (filtering of residual data outside the criterion of the median values ± 2σ). Data is plotting for individuals (filled circles); per loop-group as a boxplot (outliers indicated by stars) and the moving median values represented by a continuous line across the loops.


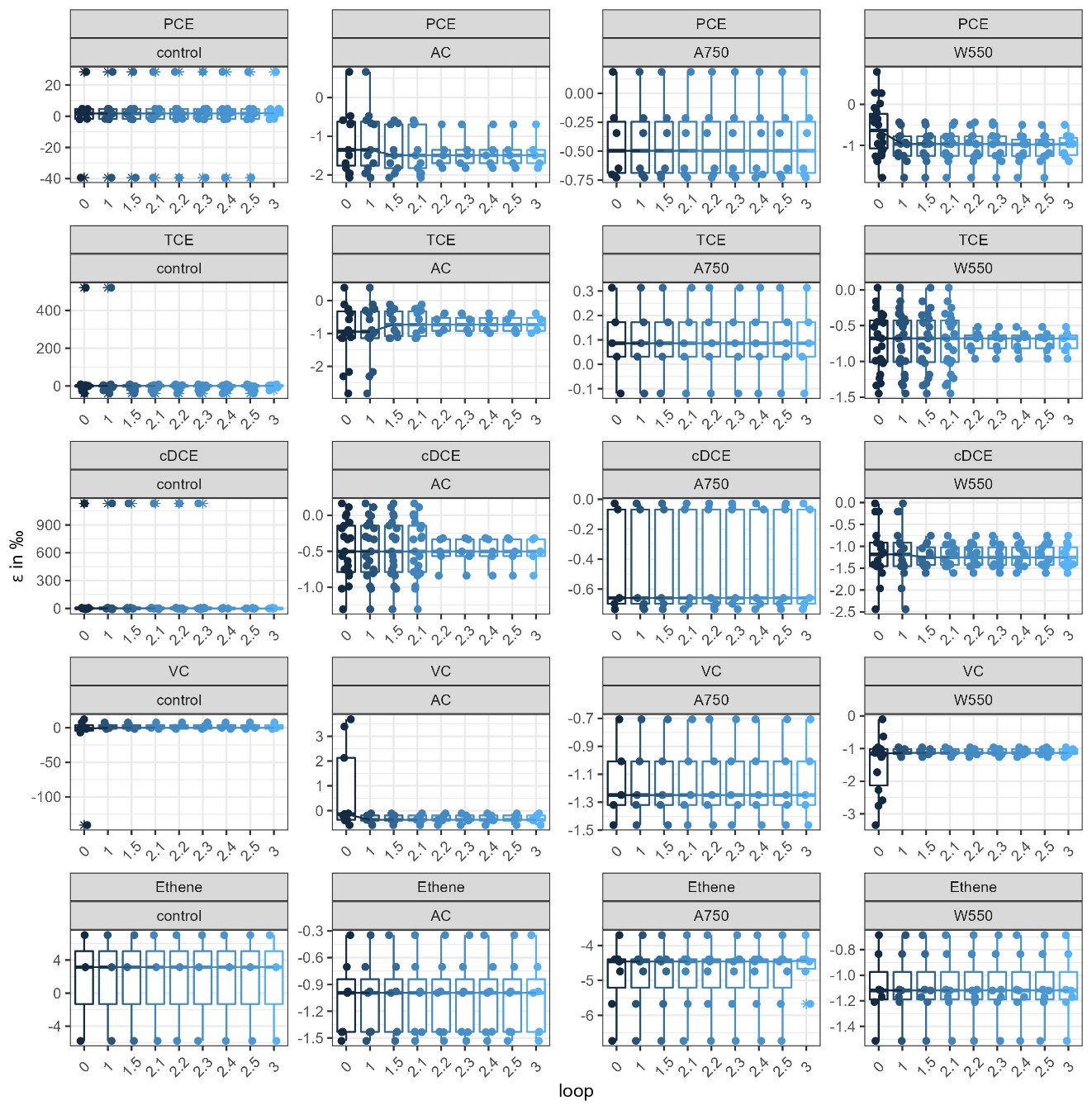


Figure A.8: Carbon isotope fractionation factors (ε in ‰) of ETs (PCE, TCE, cDCE, VC, ethene) obtained by isotherm experiments with the charcoals AC, W550 and A750. The x-axis shows the continuous filtering of raw data sets of ∆δ^13^C values (loop=0) to the final data sets (loop=3), which then obtained the shown values of ε_C_. Loops 0-3 represent the processing steps 1 (exclusion of isotherm experiments, which failed due to sampling and measurement issues), 1.5 (filtering of data sets within the median ± 1 σ), 2.1 – 2.5 (Grubbs outlier tests at a significance level α<0.05 and a minimum number of ∆δ^13^C above n=6 to check for 2 outliers on one side (2.1); one outlier on each side (2.2); single outlier for the maximum/minimum difference from the mean (2.3, 2.4) and after the iterative outlier tests (2.5)) and 3 (filtering of residual data outside the criterion of the median values ± 2σ). Data is plotting for individuals (filled circles); per loop-group as a boxplot (outliers indicated by stars, which deviate more than 3*IQR (inter-quantile range)) and the moving median values represented by a continuous line across the loops.


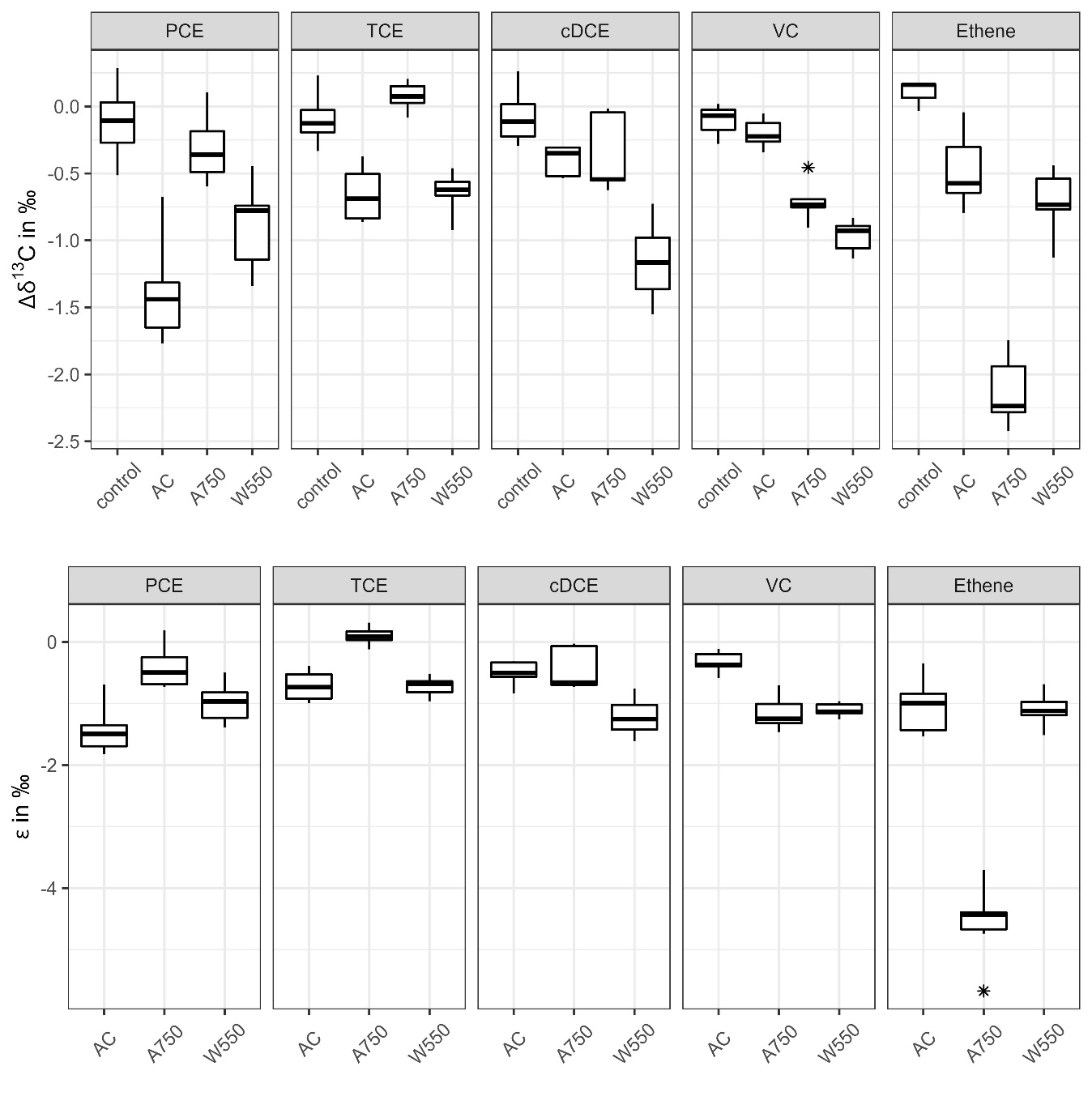


Figure A.9: Carbon isotope difference (shift in δ^13^C values from t=0 to t=equilibrium, denoted as ∆δ^13^C and carbon isotope fractionation factors (ε in ‰)) calculated from the processed data of the sorption isotherm experiments for the chlorinated ethenes PCE, TCE, cDCE, VC and for ethene for the sorption on the charcoals AC, W550, A750 and the charcoal-free controls. Data is depicted in box plots with outliers depicted outside the limit of three times the inter-quantile range.


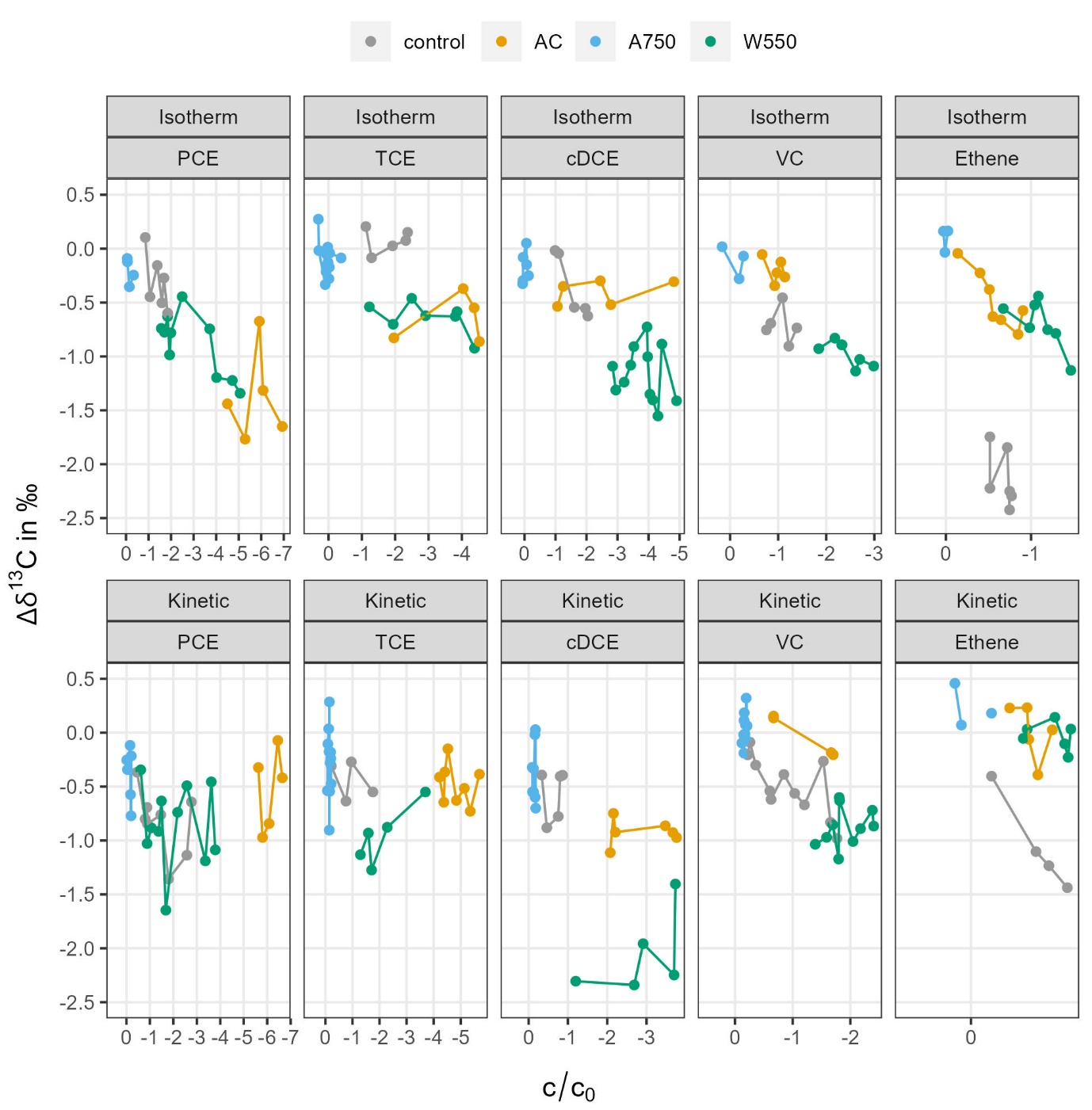


Figure A.10: Overview of carbon isotope difference (∆δ^13^C in ‰ = δ^13^C_t=0_ - δ^13^C_t=t_) versus change in concentration of ETs (PCE, TCE, cDCE, VC, ethene) over time on a log(n)-scale (c/c_0_). Top: Data from the Isotherm experiments with a single point from each sorption flask at the equilibrium. Bottom: Data from the sorption kinetic experiments with points of different instants of time from t>0 to t=equilibrium. Data point colour reflect controls and the sorbents AC, A750 and W550.
